# Study of Depression and Its Associated Factors among Women Living with HIV/AIDS in Coastal South India

**DOI:** 10.5402/2012/684972

**Published:** 2012-06-21

**Authors:** B. Unnikrishnan, Vinita Jagannath, John T. Ramapuram, B. Achappa, D. Madi

**Affiliations:** ^1^Department of Community Medicine, Kasturba Medical College, Manipal University, Light House Hill Road, Mangalore 575001, India; ^2^Department of Medicine, Kasturba Medical College, Manipal University, Light House Hill Road, Mangalore 575001, India

## Abstract

*Background*. Depression is one of the most prevalent psychiatric diagnoses seen in HIV-positive individuals. Women with HIV are about four times more likely to be depressed than those who are not infected. *Aims*. To assess the sociodemographic and clinical correlates of depression among women living with HIV/AIDS. *Setting and Design*. One public and one private hospital in Mangalore, Coastal South India, and cross-sectional design. *Methods and Materials*. Study constituted of 137 HIV-positive women, depression was assessed using BDI (Beck Depression Inventory), and social support was assessed using Lubben Social Network Scale. *Statistical Analysis*. All analysis was conducted using SPSS version 11.5. Chi-square test with *P* value less than 0.05 was taken as statistically significant. *Results*. Among 137 HIV-positive women, 51.1% were depressed. Around 16% were having moderate to high risk for isolation. Depression was statistically significant in rural women, widowed women, and lower socioeconomic class women. *Conclusion*. Depression is highly prevalent among women living with HIV which is still underdiagnosed and undertreated, and there is a need to incorporate mental health services as an integral component of HIV care.

## 1. Introduction

The acquired immuno deficiency syndrome (AIDS) is the most dreadful epidemic that mankind has ever witnessed. Depression is a common psychiatric illness characterized by low mood, decreased interest, and anergia along with a plethora of other vegetative and cognitive symptoms. Depression is a frequent consequence of trying to cope with chronic illness. Depressive symptoms in HIV positive women are associated with impaired adherence to antiretroviral therapy (ART), higher HIV plasma viral loads, higher mortality, less social support, and a worse quality of life. In resource poor setting, the burden of depression among HIV-positive people is more [1]. Major depressive disorder is four times higher in HIV-positive women (19.4%) than in HIV-negative women (4.8%) [2]. Women acquiring HIV infection is on rise these days. According to UNAIDS 2009 HIV estimates, women more than 15 years of age living with HIV in India are 730,000–1,000,000 and number of people living with HIV in India are 2,100,000–2,800,00 [3]. In India, married women are not considered as high-risk group for contracting or transmitting HIV as sexual relationships are considered to be monogamous in marriage. However, many studies suggest that married women are at risk of acquiring HIV [4–6]. HIV positive women face violent domestic attacks, financial constraints, rejection by family, spouses and community, compromised health care seeking, child care problems, emotional and mental health problems [7, 8]. Lack of social support from the community and health care staff has generated anxiety and fear among HIV-positive women, ultimately leading to depression. Depression impairs immune function thereby altering the course of HIV infection [9]. It is seen that social support is inversely related to depression [10].

In this study, we have made an attempt to assess the sociodemographic and clinical correlates of depression among HIV-positive women. This in turn would help in designing programmes for prevention and treatment of HIV-positive women.

## 2. Materials and Methods

### 2.1. Study Design

This was a cross-sectional study involving HIV-positive women between the age group of 20 to 60 years who were undergoing treatment at a tertiary level teaching private hospital (Kasturba Medical College, Attavar, Mangalore) and a public hospital in Mangalore, Coastal South India.

### 2.2. Sample and Setting

The sample size was calculated based on expected proportion of depression among HIV-positive women based on previous studies as 60%, taking relative precision as 15% and confidence interval of 95%, adding 20% as nonresponse error, the sample size was found to be 137 HIV-positive women. HIV-positive women who came to the hospital for treatment, by order of arrival, were invited to participate in the interview. It was clarified to them that this procedure would not affect their scheduled consultations.

The procedure was clearly explained to them in their local language, and all aspects of confidentiality were reassured. Those who gave written informed consent participated in the study. The interviews were conducted in a separate medical consultation room and were performed by the main researcher along with infectious disease specialist; interview lasted for about 30 minutes. In the case of a patient feeling tired or uncomfortable, she was allowed to take a break following which she could resume. Women who were found to be depressed were referred to psychiatrist for further management. The study was approved by Institutional Ethics Committee.

A pretested semistructured questionnaire was used to collect the data. Socioeconomic status was assessed using modified Kuppuswamy scale [11] which has three parameters, namely, income, occupation and educational status. The socioeconomic classes of upper, upper middle and lower middle were grouped as middle class and upper lower and lower as lower class.

BDI-Beck Depression Inventory was used to know the severity of depression, and Lubben Social Network Scale was used to assess social support. All HIV-positive women diagnosed with depression were referred to the psychiatrist's clinic later, and appropriate treatment was provided individually.

**BDI-Beck Depression Inventory [12]**

The Beck Depression Inventory (BDI) is a series of questions developed to measure the intensity, severity, and depth of depression in patients with psychiatric diagnoses. Its long form is composed of 21 questions, each designed to assess a specific symptom common among people with depression (see Table 1).

**Lubben Social Network Scale [13]**

It is a self-report 10-items scale to assess the level of social support available to a person. Each item is rated from 0 to 5. It has five domains including family network, friends' network and confidant relationship, helping others, and living arrangements. The minimum score is 0 and maximum is 50, higher scores indicating greater level of social support (see Table 2).

### 2.3. Statistical Analysis 

All analysis was conducted using SPSS (Statistical Package for Social Sciences) version 11.5. Chi-square test with *P* value less than 0.05 was taken as statistically significant. 

## 3. Results

As seen in Table 3, this study included 137 subjects. Out of this, 72 (52.6%) were from rural area and 65 (47.4%) were from urban area. Rural women were more depressed than urban women, and it was statistically significant. Majority of the subjects (83.2%) were middle aged, that is, 30–50 years which is productive population. 60.6% women were married, 23.4% were widowed, and 16% were single. It was found that widowed women were more depressed than married and single women, and it was statistically significant. Hindus constituted 80.3% followed by Christians (14.6%) and Muslims (5.1%). Around 35% belonged to middle class 65% belonged to lower class, lower class women were more depressed than upper class women, and it was found to be statistically significant. Majority of the women (80.3%) acquired the infection through sexual route, 13.9% acquired through blood, and remaining (5.8%) acquired by other routes of transmission. Women on ART constituted 36.5%, and 63.5% were not on ART. Women with CD4 count more than 250 were 64.2%, and CD4 count less than 250 were 35.8%. 

Table 4 shows 51.1% were depressed and 48.9% were not depressed. 16% of the women had moderate to high risk for isolation, and 84% of the women had low risk for isolation. 

As shown in Table 5, 41.6% women who had low risk for isolation were depressed, 9.5% women who had moderate to high risk for isolation were depressed. There was no statistically significant association between depression and social support. 

## 4. Discussion 

In this study, 137 HIV-positive women were studied as shown in Table 3. As seen in Table 3, a statistical significant association was found between depression and residence, that is, rural women were found to be more depressed than urban women. This is because rural women are isolated from social services and health care services because of lack of transportation and conservative society in rural areas which forces them not to disclose their HIV status thus depriving them of the social support and ultimately leading to depression [14]. Lower class women and widowed women were found to be more depressed than middle class women and married and single women, respectively, and it was found to be statistically significant. Financial burden and illiteracy make lower socioeconomic status more prone for depression [15]. Widow/widower does not get support from their family and are worried about the future of their kids and are more prone for depression. In a study, widows were at high risk for depression [16]. A study showed that marriage provides a social support system, and married people are less prone for depression [17]. Majority of the women (80.3%) acquired the infection through sexual route, 13.9% acquired through blood and remaining (5.8%) acquired by other routes of transmission. In our country, professional blood donation is banned since January 1 1998. Since then blood donation is voluntary. Blood is procured from NACO (National AIDS Control Organization) accredited blood banks where blood is screened for five diseases, namely HIV, hepatitis B, hepatitis C, syphilis and malaria. These are the steps taken by NACO to reduce transfusion transmitted infections [18]. 

As seen in Table 4, depression as assessed by Beck Depression Inventory (BDI) was found to be 51.1%. Many studies have shown higher rates of depression among HIV-positive women than HIV-negative women [19, 20]. 16% of the women had moderate to high risk for isolation as social support assessed by Lubben Social Network Scale. A study showed that social support plays an important role in women's health [21]. 

As shown in Table 5, it was found that there was no statistically significant association between depression and social support. In Indian setting, women get very good social support because of closely knit family system and spouse support, this may be the reason for not finding association between social support and depression. On the contrary, many studies show that social support has protective influence on depression [22–24]. 

## 5. Conclusion 

The present cross-sectional study is an attempt to elicit information among HIV-positive women. Depression in HIV-women was found to be 51.1%. Depression was statistically significant in rural women, widowed women, and lower socioeconomic class women. Thus, depression is highly prevalent among women living with HIV which is still underdiagnosed and undertreated, and there is a need to incorporate mental health services as an integral component of HIV care. 

## Figures and Tables

**Table 1:** 

Score	Severity of depression
0–9	Normal
10–16	Mild depression
17–29	Moderate depression
30–63	Severe depression

**Table 2:** 

Score	Risk of isolation
<20	Isolated
21–25	High risk for isolation
26–30	Moderate risk for isolation
≥31	Low risk for isolation

**Table 3:** Baseline characteristics of study population (*n* = 137).

Characteristics	BDI score		*P* value^∗^
	Not depressed *N* (%)	Depressed *N* (%)
Residence			
Urban	43 (66.2%)	22 (33.8%)	<0.001
Rural	24 (33.3%)	48 (66.7%)	
Marital status			
Single	15 (68.2%)	7 (31.8%)	<0.001
Married	46 (55.4%)	37 (44.6%)	
Widowed	6 (18.8%)	26 (81.3%)	
Religion			
Hindu	52 (47.3%)	58 (52.7%)	0.54
Muslim	3 (42.9%)	4 (57.1%)	
Christian	12 (60 %)	8 (40%)	
Mode of transmission			
Sexual	55 (50%)	55 (50%)	0.417
Blood	7 (36.8%)	12 (63.2%)	
Other	5 (62.5%)	3 (37.5%)	
Time since diagnosis			
<5 years	39 (43.8%)	50 (56.2%)	0.075
>5 years	28 (58.3%)	20 (41.7%)	
Antiretroviral treatment (Art)			
On art	25 (50%)	25 (50%)	0.493
Not on art	42 (48.3%)	45 (51.7%)	
Socioeconomic status (Ses)			
Middle Ses	36 (75%)	12 (25%)	<0.001
Lower Ses	31 (34.8%)	58 (65.2%)	
CD4 group			
<250	27 (55.1%)	27 (44.9%)	0.183
>250	40 (45.5%)	48 (54.5%)	

^∗^ *P* value less than 0.05 taken as statistically significant.

**Table 4:** Assessment of depression and social support.

Parameters	*N*	%
Depression		
Not depressed (^∗^BDI score 0–9)	67	48.9%
Depressed (^∗^BDI score 10–63)	70	51.1%
Social support		
High risk for isolation (^#^LSNS score 21–25)	15	10.9%
Moderate risk for isolation (^#^LSNS score 26–30)	7	5.1%
Low risk for isolation (^#^LSNS score ≥31)	115	84%

^∗^BDI: Beck Depression Inventory.

^#^LSNS: Lubben Social Network Scale.

**Table 5:** Comparison of depression and social support.

Parameters	Depression		*P* value^∗^
	Not depressed *N* (%)	Depressed *N* (%)
Social support			
High risk for isolation	6 (40%)	9 (60%)	0.71
Moderate risk for isolation	3 (42.9%)	4 (57.1%)	
Low risk for isolation	58 (50.4%)	57 (49.6%)	

^∗^ *P* value less than 0.05 taken as statistically significant.
